# The Concept of Death and Deceased Organ Donation

**Published:** 2010-02-01

**Authors:** F. L. Delmonico

**Affiliations:** *Professor of Surgery, Harvard Medical School, Massachusetts General Hospital Transplant Center, Boston, MA, USA*

**Keywords:** Dead, ethics, brain death, transplant

## Abstract

An individual who has sustained either irreversible cessation of circulatory and respiratory functions, or irreversible cessation of all functions of the entire brain, including the brainstem is dead. A person is not dead unless his brain is dead. The time-honored criteria of stoppage of the heartbeat in circulation are indicative of death only when they persist long enough for the brain to die. Brain death does not require every brain cell to be nonviable but the criteria require an irreversible loss of neurologic function of a patient interminably supported by a mechanical respirator.

For death to be diagnosed by an irreversible cessation of circulation and respiration an absence of circulation should be observed for at least two but no more than five minutes. Irreversibility is determined by a “permanent” loss of function meaning that the function will not be restored 1) because it will neither return spontaneously, nor 2) will it return as a result of medical intervention because physicians have decided not to attempt resuscitation.

## INTRODUCTION

A definition of death was established in the United States in 1980 by the National Conference of Commissioners on Uniform State Laws that formulated the Uniform Determination of Death Act (UDDA) [1]. The UDDA states that “an individual who has sustained either irreversible cessation of circulatory and respiratory functions, or irreversible cessation of all functions of the entire brain, including the brainstem is dead.” This definition was approved by the American Medical Association in 1980 and by the American Bar Association in 1981 [2]. Today, all 50 states and the District of Columbia follow the UDDA as a legal and medical standard of death.

The UDDA criteria for brain death assess the function of the entire brain—both cerebral and brainstem. The conceptual significance of assessing brainstem function is to assure that an individual breathing spontaneously is not declared dead. In the original definition of irreversible coma by the *ad hoc* Harvard Committee in 1968, the concept included an absence of spontaneous respiration [3]. 

My personal interest in this topic dates back to a period of medical school education that culminated in a project analyzing the opinion of physicians regarding the concept of death [4]. At that time, the concept of death was in transition and controversial, but there was clear leadership from individuals such as pioneering transplant surgeon, Dr. David Hume. Doctor Hume wrote “there is only one definition of death, irreversible brain damage. Cessation of heartbeat does not constitute death unless it has caused irreversible brain damage. To diagnose irreversible brain damage there must be no spontaneous respirations.” (Personal communication) 

These observations were later corroborated by Dr. William Sweet published in the *New England Journal of Medicine* when he wrote “it is clear that a person is not dead unless his brain is dead. The time-honored criteria of stoppage of the heartbeat in circulation are indicative of death only when they persist long enough for the brain to die” [5].

More recently, Dr. Sam Shemie has clarified the paradigm for donation and death by emphasizing on the “required absence of circulation” (as stipulated by the UDDA; and thus, not just heartbeat) and by underscoring the vital function of the brain as an essential criterion of life [6]. “Where extracorporeal machines or transplantation can support or replace the function of organs such as the heart, lung, liver or kidney, the brain is the only organ that cannot be supported or replaced by medical technology.”

## CHALLENGING THE CONCEPT OF DEATH AS DETERMINED BY EVALUATING NEUROLOGIC FUNCTION

Byrne and others have rejected brain death as constituting death of the person contending that “cessation of the entire brain function, whether irreversible or not, is not necessarily linked to total destruction of the brain or the death of the person” [7]. Byrne, apparently, bases his opinion regarding death as philosophically constituting a separation of the soul from the body. However, applying that personal philosophy to the diagnosis of death defies a legal and medical standard, and an ethical and practical sensibility. No one knows when the soul may separate from the body at the time of death. However, the legal and medical definition of death is clear in terms of neurologic and circulatory function. It becomes unethical to impose futile clinical treatments to a comatose individual, if the function of the entire brain is irreversibly lost. What would opponents of the brain death determination do with a patient on a ventilator with such a clinical condition—have them maintained indefinitely in such a state? To propose the brain death criteria as constituting death was the central issue that confronted the Harvard Committee in 1967. No one knows when the soul separates from the body, but a precise time of death must be specified for obvious legal, medical and social reasons, so that futile treatment can be concluded (without further obligation or responsibility to provide resuscitative or supportive technologies) and proper disposition of the body with burial and estate and property transfer, *etc* can be exercised [6]. 

For many years, Truog has also objected to the determination of death by neurologic evaluation and by circulatory function [8]. He recently wrote in the *New England Journal of Medicine* that “arguments about why these patients should be considered dead have never been fully convincing. The definition of brain death requires a complete absence of all functions of the entire brain yet many of these patients retaining essential neurologic function, such as regulated secretion of hypothalamic hormones” [9]. The rebuttal to this assertion has been given by Shemie who claimed that “the release of antidiuretic hormone (ADH) from the hypothalamus is not considered to be essential neurologic function; rather, neurologic function is determined by an absence of consciousness, receptivity and responsiveness, spontaneous movement, spontaneous breathing, and an absence of brainstem reflexes.” (Personal communication)

Brain death does not require every brain cell to be nonviable but the criteria require an irreversible loss of neurologic function of a patient interminably supported by a mechanical respirator. For Truog and Shewmon however, these patients are not considered dead because they indeed can be supported indefinitely beyond the acute phase of their illness [10]. It is well known however, that despite the irreversible loss of brain function, the remainder of the body can be maintained by mechanical support; for example, even by patients who become brain-dead during pregnancy yet successfully have their fetuses brought to term. The clinical condition still constitutes the death of the mother and a viable fetus by continued mechanical support until birth [6]. 

## CHALLENGING THE CONCEPT OF DEATH AS DETERMINED BY ABSENCE OF CIRCULATION

Again, in the *New England Journal of Medicine*, Truog and Veatch have recently asserted that donation after cardiac death (DCD) is not acceptable; that is, the recovery of organs after the determination of death by circulatory and respiratory criteria [9, 11]. Troug suggests that the recovery of the heart following DCD is “paradoxical” because “the heart of patients who have been declared dead on the basis of the irreversible loss of cardiac function have in fact been transplanted and successfully functioned in the chest of another.” Veatch is similarly not convinced that the donor is dead and stated that “if someone is pronounced dead on the basis of irreversible loss of heart function, after all, it would not be possible for heart function to be restored in another body” [11]. 

Both Veatch and Troug misinterpret the UDDA which precisely stated that it applies to an individual who has sustained irreversible cessation of circulatory and respiratory functions. It is not a matter of the cessation of heartbeat or cardiac function *per se* but an irreversible cessation of circulation in the donor. The consequence of the absence of circulation is upon the function of the brain. An irreversible loss of blood flow to the brain results in an irreversible loss of neurologic function—the UDDA definition of death. 

Bernat has written that circulation—not heartbeat—is the critical function that must be lost using circulatory-respiratory tests to determine death [12]. For example, we do not declare patients dead who are on heart-lung machines during cardiac surgery, on ECMO awaiting heart transplantation (even if they never receive a heart), or carrying artificial hearts because, despite absence of heartbeat, their circulation remains continuously maintained. That is why the death standard requires absence of circulation.

“Whether the asystolic heart is subsequently left alone, removed and not restarted, or removed and restarted in another patient is irrelevant to the circulatory status of the just-declared dead patient. Removing and restarting the heart elsewhere simply has no impact on the previous death determination because that patient remains permanently without circulation in exactly the same way as if the non-beating heart had been left in place.” (Bernat’s personal communication)

## DEFINING CESSATION AND IRREVERSIBILITY

For the determination of death by the irreversible cessation of circulatory and respiratory functions in a controlled setting of organ donation, that is, following the withdrawal of futile treatment-controlled DCD in a hospital setting, cessation and irreversibility should be defined. Cessation is recognized by clinical examination that detects the absence of responsiveness, heart sounds, pulse and respiratory effort [13]. The medical circumstances of DCD may require the use of confirmatory objective tests such as electronic monitoring or absence of pulse pressure as determined through an arterial catheter. 

Bernat has introduced the concept of permanency to confirm irreversibility and proposed the following formulation: An “irreversible” loss of function means that the function cannot be restored by any known technology [13]. Bernat stated that “irreversibility” is an absolute and univocal condition that implies impossibility and does not rely on intent or action. In contrast, a “permanent” loss of function means that the function will not be restored because it will neither return spontaneously, nor will it return as a result of medical intervention—because physicians have decided not to attempt resuscitation. “Permanent” is a contingent and equivocal condition that admits possibility and relies on intent and action. The two conditions are causally related. All functions that are irreversibly lost are also permanently lost (but not *vice versa*) and in DCD death determinations, functions that are lost permanently, quickly and inevitably become lost irreversibly. The National Conference on Donation after Cardiac Death accepted this formulation that the irreversible loss of circulation is confirmed by the observation that circulation will not resume spontaneously and circulation will not be restored on medically and ethically justifiable grounds.

Irreversibility is recognized by persistent cessation of functions during an appropriate period of observation [13]. 

## THE DURATION OF ABSENCE OF CIRCULATION

Such criteria now include a period of witnessing the cessation of circulation by the patient care team (independent of the organ recovery or transplant team) of at least two and no more than five minutes following the initial observation of asystole to attest its irreversibility.

## THE USE OF EXTRACORPOREAL MACHINE OXYGENATION (ECMO)

Protocols administering ECMO to the donor after the determination of cardiac death have become controversial. Bernat has written a companion article in the *New England Journal of Medicine* specifically addressing the issue of ECMO [12]. He noted that “if ECMO adequately provided circulation and oxygenation to the donor’s entire body, it would retroactively negate the death determination by preventing the loss of circulation and respiration from becoming permanent or irreversible, potentially ‘reanimating’ the heart and preventing the progression to brain destruction on which the circulatory criterion of death is predicated.”

These protocols attempt to circumvent heart reanimation or resumption of brain circulation by the placement of an inflatable balloon catheter in the aorta. There is general agreement that restoring circulation by ECMO following the declaration of death by any reversible cessation of circulation is fundamentally contradictory to the death declaration. To accomplish a declaration of death by absence of neurologic function in this setting by either the insertion of a balloon catheter in the thoracic aorta or the ligation of the carotid artery is unacceptable because it becomes the active causation of absence of neurologic function. If that approach was to be ethically permissible, the rhetorical question might be posed: Why not ligate the carotid arteries antecedent of the spontaneous absence of circulation?

## THE DENVER PROTOCOL

Boucek, *et al*, have presented case reports in the *New England Journal of Medicine* about the recovery of hearts from infants declared dead by absence of circulation and stated that “when cardiocirculatory function ceased, the first patient was observed for three minutes before death was declared and the organ-donation process initiated. On the basis of recommendations of the ethics committee, for the other two donors, the observation period was shortened to 1.25 minutes” [14]. 

Bernat has criticized this approach and mentioned “what minimum duration of asystole ensures that autoresuscitation will not occur is an empirical question that can be answered conclusively only after observing many hundreds of patients” [12]. The Institute of Medicine and the Canadian Council for Donation and Transplantation recommend a period of five minutes of absent circulation to elapse before the patient is declared dead (15). In 2005, participants in a national conference on this topic agreed with the recommendation by the Society of Critical Care Medicine to wait at least two and at most five minutes [16]. 

## THE USE OF HEPARIN

The administration of heparin at the time of the withdrawal of life sustaining treatment is the current standard of care and a key component of DCD best practice. The long-term survival of the transplanted organ may be at risk if thrombi impede circulation to the organ after reperfusion. The omission of heparin could negatively impact organ recovery and hinder the acceptance of recovered organs for transplantation. 

The use of heparin has been considered controversial on the basis of theoretical concerns that it may hasten the death of the donor [17]. However, there is no evidence that heparin would cause sufficient bleeding after the withdrawal of treatment to be the cause of death. While heparin may prevent clotting in a patient who is actively bleeding, it is unlikely to cause bleeding in a head injured patient who is not actively bleeding. It should not be overlooked that the event of demise is the withdrawal of life support that affects the loss of circulation and respiration (and not the use of heparin). 

Finally, the principle of double effect asserts that an action that produces a good effect and a bad effect might be permissible if the good effect is intended and the bad effect is merely foreseen but unintended.

## THE DEAD DONOR RULE AND ORGAN DONATION

Robertson wrote more than a decade ago that the retrieval of organs for transplantation should not cause the death of a donor [18]. This rule has since been the ethical axiom of organ donation; thus, no organ recovery should precede the declaration of death. 

Recently however, Truog has stated that “a better approach to procuring vital organs while protecting vulnerable patients against abuse would be to emphasize the importance of obtaining valid informed consent for organ donation from patients or surrogates before the withdrawal of life-sustaining treatment in situations of devastating and irreversible neurologic injury” [9].

There is no support or intention of the organ donation community to rescind the dead donor rule. The public trust in organ donation hinges upon an assurance that the medical professional will prioritize the care of the dying patient over any other objectives, however noble or good. 

One could readily anticipate a societal skepticism if medical professionals present the following approach to the family of a dying patient; it was described by Troug that “your family member has a devastating neurologic injury but is not dead. If you consent to the removal of organs now before the determination of death, it will result in the death of your family member but it would enhance the possibility of successful transplantation of organs.” The rejection of that scenario as proposed by Troug, is evident in the recent trial of a transplant surgeon that was accused of hastening the death of an individual in the recovery of organs [19]. 

## CHURCH POSITION

In the address of Pope John Paul II to the Transplantation Congress in Rome in 2000, regarding the determination of death, he said “… it is helpful to recall that the death of the person is a single event, consisting in the total disintegration of that unitary and integrated whole that is the personal self.” And that “it is a well-known fact that for some time certain scientific approaches to ascertaining death have shifted the emphasis from the traditional cardiorespiratory signs to the so-called ‘neurological’ criterion. Specifically, this consists in establishing, according to clearly determined parameters commonly held by the international scientific community, the complete and irreversible cessation of all brain activity (in the cerebrum, cerebellum and brain stem). This is then considered the sign that the individual organism has lost its integrative capacity” [20].

For those who are involved in the transplantation of organs from the deceased, this Papal testimony is reassuring of a moral propriety that can be defended medically—the valid concept of death by neurologic criteria [21]. 
